# The Effect of GB1 on DSS-Induced Colitis in WT and Nlrp3^-/-^ Mice

**DOI:** 10.3390/ijms26094016

**Published:** 2025-04-24

**Authors:** Ziyi Zhou, Lixian Wang, Ruhe Liao, Qin Chen, Changhui Liu, Jianping Song, Changsheng Deng, Xinan Huang

**Affiliations:** 1 Artemisinin Research Center, Guangzhou University of Chinese Medicine, Guangzhou 510405, China; ziyeechou@163.com (Z.Z.); wanglixian2022@126.com (L.W.); lrh2021009@163.com (R.L.); cleo0806@163.com (Q.C.); songjp@gzucm.edu.cn (J.S.); 2 School of Pharmaceutical Sciences, Guangzhou University of Chinese Medicine, Guangzhou 510405, China; liuchanghui@gzucm.edu.cn; 3 State Key Laboratory for Quality Ensurance and Sustainable Use of Dao-di Herbs, Beijing 100700, China

**Keywords:** Garcinia biflavonoid 1, ulcerative colitis, NLPR3^-/-^ mice, inhibiting NLRP3

## Abstract

This study investigated the protective effects of Garcinia biflavonoid 1 (GB1) against dextran sulfate sodium (DSS)-induced ulcerative colitis and its underlying mechanisms. Using wild-type (WT) and NLRP3 knockout (Nlrp3^-/-^) mice, we demonstrated that GB1 administration significantly ameliorated colitis symptoms, as evidenced by improved body weight, disease activity index (DAI) scores, colon length, and histological damage in WT mice. Mechanistically, GB1 downregulated pro-inflammatory mediators (IL-6, NF-κB, and CD11b) while attenuating the expression of NLRP3 inflammasome components (ASC, Caspase-1, and IL-1β). Notably, these protective effects were abolished in Nlrp3^-/-^ mice, confirming the essential role of NLRP3 in GB1-mediated mitigation of colitis. Furthermore, GB1 reinforced intestinal barrier integrity by preserving tight junctions, reducing permeability, and attenuating mucosal inflammation. Collectively, our findings highlight GB1 as a promising therapeutic candidate for colitis treatment, primarily through NLRP3 inflammasome suppression and intestinal barrier restoration.

## 1. Introduction

Inflammatory bowel disease (IBD), encompassing ulcerative colitis (UC) and Crohn’s disease (CD), is a chronic gastrointestinal disorder characterized by recurrent inflammation and an elevated risk of colorectal cancer, posing a substantial global health burden [1,2]. While UC was historically prevalent in Western nations, its incidence has risen worldwide, underscoring its growing public health significance.The pathogenesis of UC is not fully understood but is likely linked to genetic factors, environmental influences, immune dysregulation, and changes in immune cell types and quantities in the intestinal microbiota and mucosa [3,4,5]. Notably, immune dysfunction plays a pivotal role, with emerging evidence implicating the NOD-like receptor protein 3 (NLRP3) inflammasome as a key mediator. Dysregulated immune responses can disrupt intestinal epithelial barrier integrity, exacerbating mucosal inflammation and UC progression [6].

NLRP3 is essential in ulcerative colitis (UC). Clinical data indicate around 163 genetic susceptibility loci linked to UC, primarily immune-related, including the NLRP3 gene locus. Activation of the NLRP3 inflammasome increases IL-1β expression, contributing to UC prevalence and underscoring its significant role in UC pathogenesis. The inflammasome behaves differently during disease progression; normally, NLRP3 is minimally expressed, but inflammatory bowel disease triggers increased cellular permeability and intestinal mucosa damage, activating the inflammasome. Guo et al. found that DSS-induced macrophages secrete high levels of IL-1β, requiring Caspase-1 pathway stimulation for release; however, macrophages lacking NLRP3, ASC, or Caspase-1 show minimal IL-1β expression [7]. Contrarily, some studies suggest that knocking out inflammasome genes may increase UC susceptibility. Given these conflicting findings, some researchers argue that the NLRP3 inflammasome detects gut pathogens and initiates a defense mechanism, explaining why Nlrp3^-/-^ mice, lacking this function, are more susceptible to disease. Additionally, variations in chemical modeling methods may alter gut microbiota [8,9]. Further research is necessary to clarify the NLRP3 inflammasome’s mechanisms in inflammatory bowel diseases.

Tight junctions (TJs) play a pivotal role in maintaining intestinal epithelial barrier integrity by regulating selective permeability and preventing the translocation of pathogenic antigens (e.g., bacteria, viruses, and endotoxins) into systemic circulation, thereby mitigating local and systemic inflammatory and immune responses. Structurally, TJs are composed of the zonula occludens (ZO) protein family, occludin, and claudin proteins. Notably, IBD dysregulated TJ protein expression is observed, characterized by downregulation of occludin and ZO-1 and upregulation of claudin-2, which correlates with disease progression and impaired mucosal healing [9]. In the early stages of the disease, IBD patients often display abnormal expression of tight junction proteins, potentially accelerating disease progression, indicating that tight junction integrity is crucial for mucosal healing.

Current clinical management of IBD relies on aminosalicylates, corticosteroids, and immunosuppressants, yet their therapeutic efficacy remains suboptimal and is often accompanied by significant adverse effects. Consequently, the pursuit of therapeutics that demonstrate both high efficacy and safety has become one of the focal points in current IBD research.

Garcinia kola (commonly known as bitter kola or false kola) is a medicinal plant indigenous to West Africa, particularly Nigeria’s Igbo region, where its seeds have been traditionally used to treat gastrointestinal disorders such as diarrhea and dysentery. The primary bioactive constituent of Garcinia kola extract, GB1, has demonstrated significant pharmacological properties including anti-inflammatory, antioxidant, and hypoglycemic activities. Our previous studies have shown that GB1 improves lipid metabolism in HepG2 hepatocytes and exhibits antidiabetic effects in type 2 diabetic db/db mice [10]. However, the potential protective effects of GB1 against DSS-induced colonic injury and its underlying mechanisms remain unexplored. This study aims to investigate these aspects, potentially offering new therapeutic avenues for colitis treatment.

## 2. Results

### 2.1. GB1 Alleviated DSS-Induced Colon Injury in Mice

**Figure 1:**
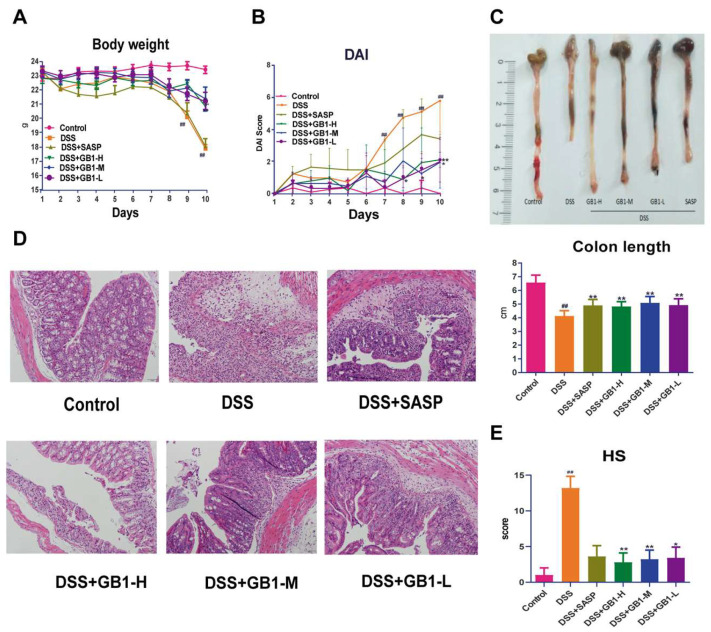
The effect of GB1 on DSS-induced colitis in mice. (**A**) The weight change of mice after the onset of DSS-induced colitis; (**B**) The score of disease activity index; (**C**) colon length; (**D**) Pathological changes of colon tissue (HE staining, 200×); (**E**) Histological score of HE stained colon sections. ## *p* < 0.01, versus with control group, * *p* < 0.05, ** *p* < 0.01, versus with DSS group.

In our DSS-induced colitis mouse model, we systematically evaluated disease progression through multiple parameters: body weight changes, bloody diarrhea incidence, colon shortening, and inflammatory cell infiltration (Figure 1). The Disease Activity Index (DAI), incorporating weight loss, stool consistency, and rectal bleeding, was assessed daily prior to treatment administration. DSS challenge induced characteristic pathological manifestations, including pronounced weight reduction, elevated DAI scores, and significant colon shortening. GB1 pretreatment effectively mitigated these effects, demonstrating: attenuated body weight loss (Figure 1A), improved DAI scores across all treatment groups (Figure 1B), and preserved colon length compared to DSS controls (Figure 1C). Histopathological examination revealed GB1- and SASP-treated mice showed substantial mucosal protection, characterized by intact crypt architecture, diminished leukocyte infiltration, and restored goblet cell populations (Figure 1D). Quantitative histological scoring (HS) confirmed these observations, with both treatment groups exhibiting significantly lower HS values than DSS-treated animals (Figure 1E).

### 2.2. GB1 Improve the Destruction of Intestinal Barrier in Colitis

**Figure 2:**
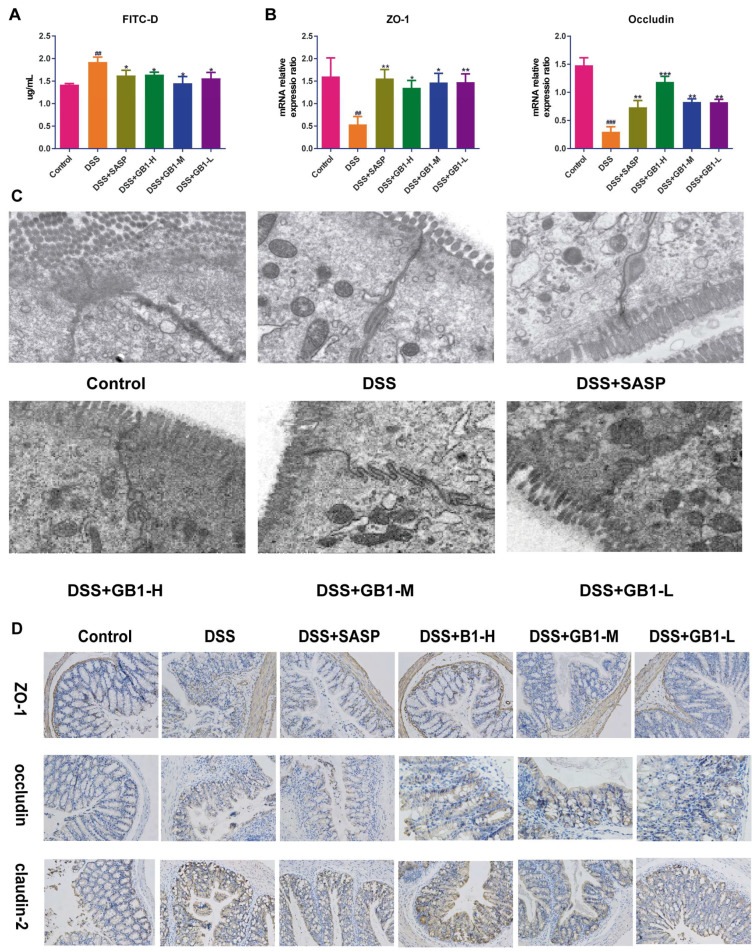
The effect of GB1 on the intestinal barrier of DSS-induced colitis mice. (**A**) Detection of colonic mucosal permeability (FITC-D) after the onset of DSS-induced colitis; (**B**) The mRNA expression of ZO-1 and Occludin in colon tissue detected by qRT-PCR; (**C**) The TJs between intestinal epithelial cells were observed by transmission electron microscopy (yellow arrows represent TJs, 10,000×); (**D**) Immunohistochemical detection of ZO-1, Occludin, Claudin-2 expression in colon tissue. ## *p* < 0.01, ### *p* < 0.001, versus with control group, * *p* < 0.05, ** *p* < 0.01, *** *p* < 0.001 versus with DSS group.

DSS administration significantly increased both the concentration and intestinal permeability of fluorescein isothiocyanate dextran (FITC-D) in our experimental model. Notably, GB1 or SASP pretreatment effectively attenuated these DSS-induced effects, demonstrating reduced FITC-D levels and improved barrier function (Figure 2A). At the molecular level, quantitative PCR analysis revealed that GB1 and SASP treatment upregulated mRNA expression of tight junction components ZO-1 and occludin in colonic tissues (Figure 2B). Ultrastructural examination by electron microscopy showed that both treatments enhanced tight junction density, reduced intercellular space, restored microvilli organization, and preserved desmosome integrity compared to DSS-treated controls (Figure 2C). Immunohistochemical analysis confirmed these findings at the protein level, with ZO-1, occludin, and claudin-2 expression patterns mirroring their respective mRNA profiles (Figure 2D).

### 2.3. GB1 Alleviates Colitis in Mice by Inhibiting the Activation of the NLPR3 Inflammasome

**Figure 3:**
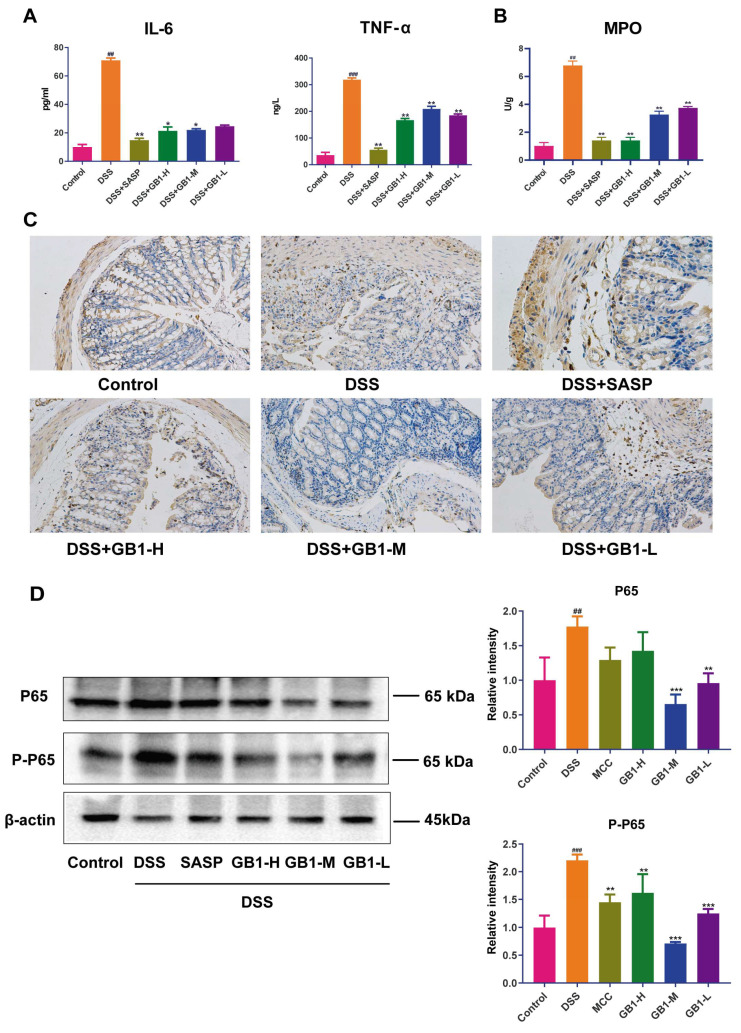
The effect of GB1 on DSS-induced colon inflammation in mice. (**A**) ELISA to determine the content of IL-6 and TNF-α in colon tissue; (**B**) Kit to detect myeloperoxidase (MPO) activity in colon tissue; (**C**) Immunohistochemical detection of CD11b expression in colon tissue (200×); (**D**) The expression levels of NF-κB p65 and P-NF-κB p65 in mouse colon tissue were detected by western blot. Versus with Control group, ## *p* < 0.01, ### *p* < 0.001; versus with DSS group, * *p* <0.05, ** *p* < 0.01, *** *p* < 0.001.

DSS administration significantly upregulated pro-inflammatory cytokines IL-6 and TNF-α in colonic tissues, while GB1 treatment effectively suppressed their production (Figure 3A). Myeloperoxidase (MPO) activity, a marker of neutrophil infiltration, was dramatically elevated in DSS-exposed tissues but significantly attenuated by GB1 or SASP pretreatment (Figure 3B). Immunoblot analysis demonstrated that both GB1 and SASP downregulated CD11b protein expression (Figure 3C). Importantly, we observed that DSS exposure activated the NF-κB pathway, as evidenced by increased total and phosphorylated p65 levels. Both GB1 and SASP pretreatment potently inhibited this DSS-induced NF-κB activation and phosphorylation (Figure 3D).

**Figure 4:**
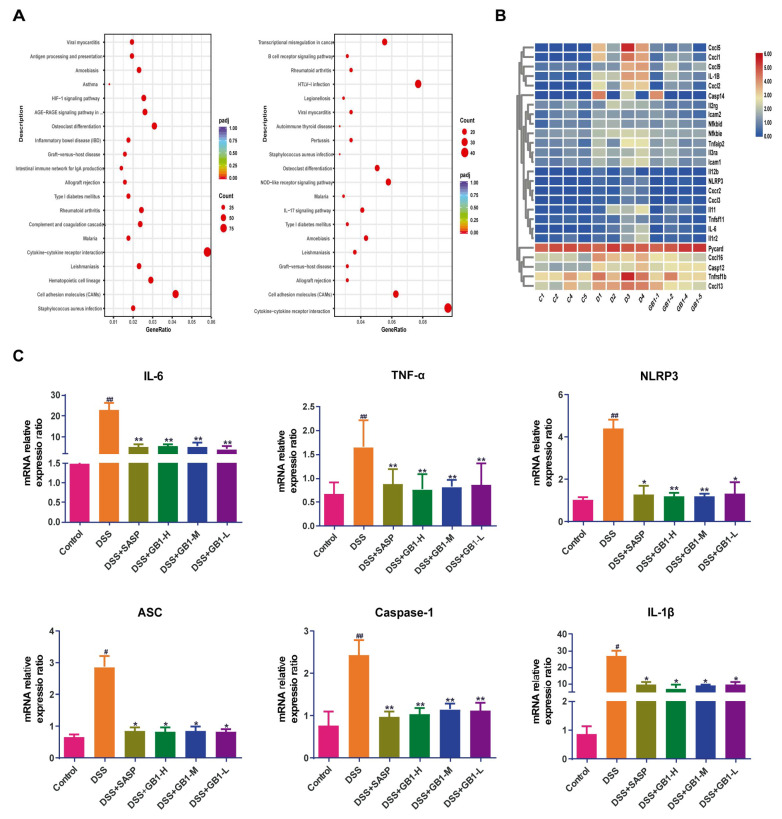
mRNA-seq experiment. (**A**) KEGG enrichment analysis (x axis represents the differential gene set, y axis represents the KEGG enrichment pathway, and the dot size represents the number of genes annotated on the KEGG pathway); (**B**) Differential gene expression levels (C represents normal group, D represents model group, GB1 represents drug group, blue represents low expression, and red represents high expression); (**C**) The mRNA expression levels of IL-6, TNF-α, NLRP3, ASC, Caspase-1 and IL-1β in colon tissue were detect-ed by qRT-PCR. # *p* < 0.05, ## *p* < 0.01, versus with control group; versus with DSS group, * *p* < 0.05, ** *p* < 0.01.

KEGG pathway enrichment analysis revealed that the cytokine-cytokine receptor interaction pathway exhibited the highest enrichment score and the largest proportion of differentially expressed genes, followed by the cell adhesion molecules (CAMs) pathway. Significant enrichment of the inflammatory bowel disease (IBD) pathway supported the modeling validation, and the NOD-like receptor signaling pathway also showed increased enrichment (Figure 4A). The heatmap illustrating differential gene expression demonstrated low expression in the normal group, high expression in the DSS group, and low expression in the GB1 group (Figure 4B). Quantitative reverse transcription polymerase chain reaction (qRT-PCR) analysis showed that mRNA levels of IL-6, TNF-α, NLRP3, ASC, Caspase-1, and IL-1β were significantly elevated after DSS induction, while GB1 pretreatment significantly reduced their expression compared to the DSS group (Figure 4C).

### 2.4. Molecular Docking Results of GB1 and NLRP3-PYD

**Figure 5:**
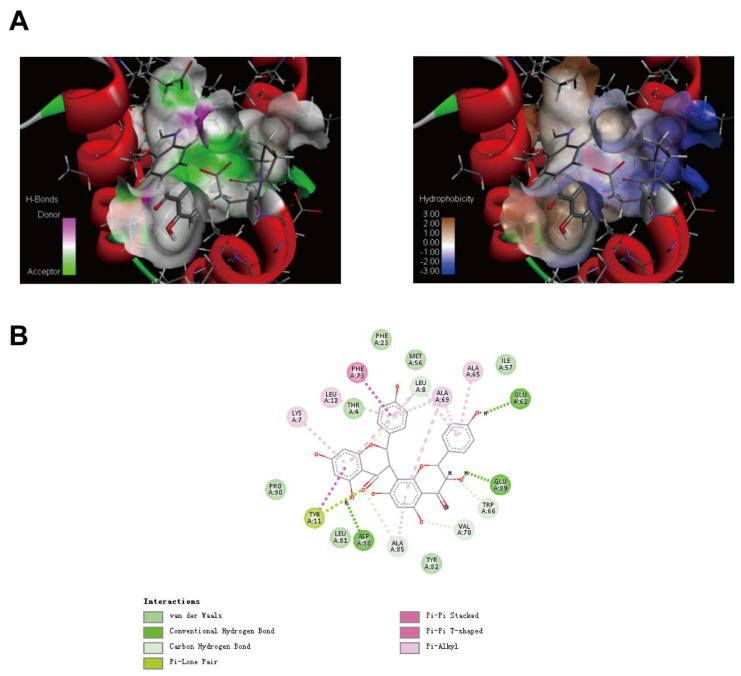
The molecular docking diagram of GB1 and NLRP3-PYD. (**A**) 3D sche matic diagram of GB1 combined with NLRP3-PYD; (**B**) 2D schematic diagram of GB1 combined with NLRP3-PYD.

3D schematic diagram of GB1 combined with NLRP3-PYD (Figure 5A). The docking score of NLRP3 protein and GB1 is more than 7 points. The total score of the target protein 2naq and GB1 is 10.4516 and CSCORE is 4 points. Discovery Studio software (https://www.3ds.com/products/biovia/discovery-studio, accessed on 19 March 2025) performs MOLCAD analysis on the docked small molecules and proteins to obtain the surface hydrophobic region of the target protein 2naq and the hydrogen bond donor and acceptor regions, as well as the binding mode map of the target protein and GB1 (Figure 5B). A Van der Waals interaction force was formed between GB1 and protein residues like ILE 57, MET 56, PHE 23, THR 4, PRO 90, LEU 81, TYR 82, and forms hydrogen bonds with protein residues GLU 62, GLU 89. A π lone pair electron interaction was formed between GB1 and protein residue TYR 11, and a π-π T-type interaction was formed between GB1 and protein residue PHE 73. In addition, GB1 forms a hydrophobic interaction with protein residues ALA 65, ALA 69, LEU 12, and LYS 7.

### 2.5. Inhibition of NLRP3 Is Indispensable of GB1 in the Protective Effects Against DSS-Induced Colitis

**Figure 6:**
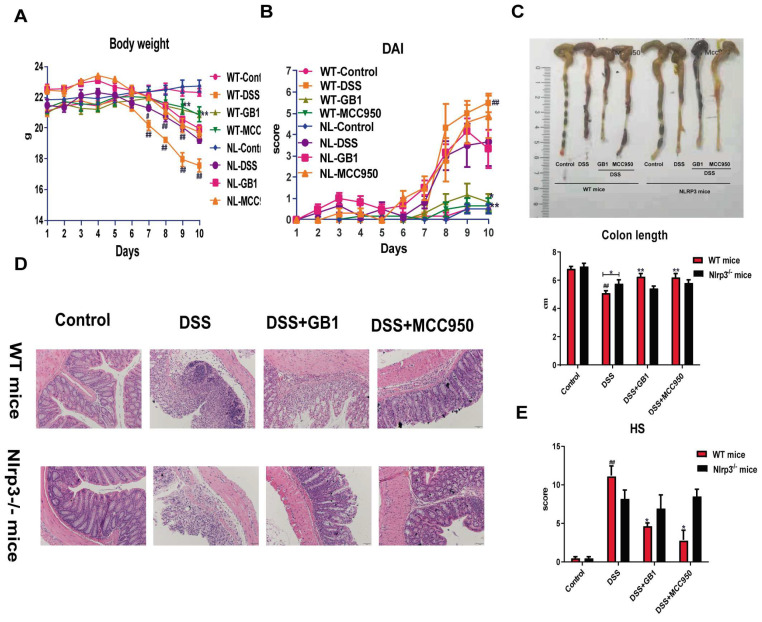
The effect of GB1 on DSS-induced colitis in WT and Nlrp3^-/-^ mice. (**A**) The weight change of WT and Nlrp3^-/-^ mice after onset of DSS-induced colitis; (**B**) The score of disease activity index of WT and Nlrp3^-/-^ mice; (**C**) Colon length of WT and Nlrp3^-/-^ mice; (**D**) Pathological changes of colon tissue of WT and Nlrp3^-/-^ mice (HE staining, 200×); (**E**) Histological score of HE stained colon sections of WT and Nlrp3^-/-^ mice. # *p* < 0.05, ## *p* < 0.01, versus with control group; * *p* < 0.05, ** *p* < 0.01, versus with DSS group.

The body weight of WT and Nlrp3^-/-^ mice decreased significantly due to colitis, with Nlrp3^-/-^ mice showing less weight loss than WT mice. In the high-dose (150 mg·kg^−1^) GB1 treatment group, WT mice had a notable weight increase compared to the DSS control group, while Nlrp3^-/-^ mice maintained stable weights (Figure 6A). DSS administration raised the DAI for both groups, but Nlrp3^-/-^ mice had a lower DAI than WT mice. WT mice experienced a substantial reduction in DAI in the high-dose GB1 or MCC950 treatment groups when compared to the DSS group, but not Nlrp3^-/-^ mice, as shown in Figure 6B. Colon morphology and length assessments indicated that both groups had significantly shorter colons than the normal control group. The colon length of treated WT mice increased after high-dose GB1 or MCC950 treatment, whereas the DSS model group did not change (Figure 6C). The colonic epithelium was preserved in both normal WT and Nlrp3^-/-^ mice, with an orderly gland arrangement and a high density of goblet cells, without any mucosal erosion or acute inflammation when stained with HE. After DSS induction, Nlrp3^-/-^ mice exhibited thickened colonic walls, structural disorganization, epithelial necrosis, glandular destruction, distorted crypts, reduced goblet cell populations, and significant inflammatory cell infiltration, while WT mice showed even more severe damage. Colonic mucosal injury in WT mice improved considerably after pre-treatment with GB1 or MCC950, as evidenced by a decrease in glandular crypt destruction and inflammatory cell infiltration, as well as an increase in goblet cells. In contrast, Nlrp3^-/-^ mice showed no alleviation of mucosal damage or inflammatory infiltration (Figure 6D). DSS induction caused a significant increase in histological score (HS), but pretreatment with GB1 or MCC950 decreased HS in WT mice compared to the DSS group, with no variations in Nlrp3^-/-^ mice (Figure 6E).

**Figure 7:**
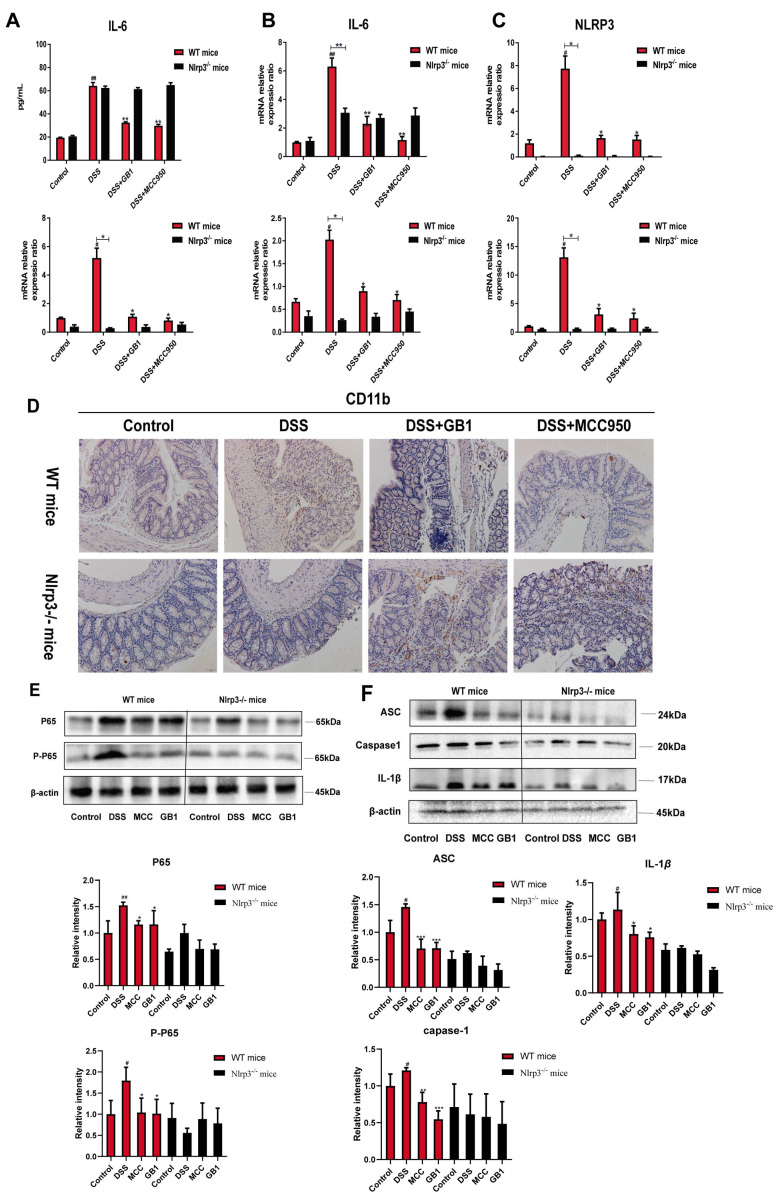
Effect of GB1 on DSS-induced colitis inflammation in mice. (**A**) ELISA kit to detect the content of IL-6 in serum; (**B**) qRT-PCR detection of IL-6 mRNA expression in colon tissue; (**C**) qRT-PCR detection of NLRP3, ASC, Caspase-1, IL-1β mRNA expression levels in colon tissue (**D**) Immunohistochemical detection of CD11b expression in colon tissue (200×); (**E**) NF-κB p65, P-NF-κB p65 protein expression levels in colon tissue were detected by western blot. # *p* < 0.05, ## *p* < 0.01, versus with control group; * *p* < 0.05, ** *p* < 0.01, versus with DSS group. The impact of GB1 on the NLRP3 signal path.; (**F**) Western blot detection of protein expression levels of ASC, Caspase-1, IL-1β in colon tissue. # *p* < 0.05, ## *p* < 0.01, versus with control group; * *p* < 0.05, ** *p* < 0.01, *** *p* < 0.001, versus with DSS group.

Serum cytokine analysis revealed that DSS challenge significantly elevated IL-6 levels in both genotypes, with Nlrp3^-/-^ mice exhibiting lower concentrations than WT controls (Figure 7A). GB1 treatment effectively reduced IL-6 secretion in WT mice, while no significant changes were observed in Nlrp3^-/-^ mice. This finding was corroborated by parallel reductions in IL-6 mRNA expression (Figure 7B). Immunohistochemical analysis demonstrated that DSS-induced upregulation of CD11b in colonic tissues was attenuated by GB1 or MCC950 pretreatment in WT but not Nlrp3^-/-^ mice (Figure 7D). Similarly, DSS-mediated NF-κB phosphorylation was significantly blunted in Nlrp3^-/-^ mice compared to WT controls. GB1 treatment suppressed NF-κB activation in WT mice without affecting obviously Nlrp3^-/-^ mice (Figure 7E).

The mRNA expression levels of NLRP3 and its downstream targets, ASC, Caspase-1, and IL-1β, were assessed. In WT mice, the mRNA levels of NLRP3, ASC, Caspase-1, and IL-1β were elevated following DSS treatment, whereas Nlrp3^-/-^ mice exhibited negligible expression. Pre-treatment with GB1 or MCC950 resulted in a significant reduction of mRNA expression in WT mice compared to the model group, with no notable changes observed in Nlrp3^-/-^ mice (Figure 7C). Additionally, the protein levels of ASC, Caspase-1, and IL-1β were markedly increased upon DSS induction, with Nlrp3^-/-^ mice showing lower protein expression than their WT counterparts. In WT mice, administration of GB1 or MCC950 led to an upregulation of these proteins, while no significant alterations were detected following NLRP3 knockout (Figure 7F).

## 3. Discussion

The incidence of ulcerative colitis (UC) has increased in China. The colon is primarily affected by UC, a recurrent, non-transmural inflammatory disease that can cause symptoms such as abdominal pain, diarrhea, rectal bleeding, and weight loss. Research into its pathogenesis is growing, while current treatments like glucocorticoids and immunosuppressants have significant side effects. Therefore, safer and more effective drugs are needed. In this study, we induced UC in C57BL/6 mice using 4% DSS and treated them with varying concentrations of GB1. DSS induction resulted in decreased mouse weight, increased DAI scores, and colon shortening. Histopathological analysis revealed disrupted tissue structure, crypt loss, and immune cell infiltration. These symptoms were reduced by GB1 pretreatment, which suggests that GB1 has an inhibitory effect on DSS-induced UC.

Preventing microbial and toxin translocation is made possible by the intestinal mucosal barrier, which includes mechanical, immune, chemical, and biological components. The mechanical barrier, primarily consisting of intestinal epithelial cells and intercellular junctions (e.g., tight junctions (TJ), adherens junctions (AJ), desmosomes, and gap junctions), is a key element. TJs are particularly significant. Following DSS induction, the intestinal epithelial tight junctions and mucosal barrier were found to be disrupted by transmission electron microscopy.

Intestinal mucosal barrier permeability was elevated, whereas GB1 treatment ameliorated the disruption of intestinal epithelial tight junctions (TJs). TJs, protein complexes localized on the apical aspect of adjacent epithelial cells, comprise transmembrane proteins (Occludin, claudins, junctional adhesion molecules [JAMs]), peripheral membrane proteins (ZOs), and cell regulatory molecules (actin, myosin, and other cytoskeletal proteins). Occludin is typically expressed on the surface and cytoplasm of intestinal epithelial and glandular cells, but it can occasionally localize to the nucleus. Occludin recruits signal transduction molecules to TJs, thereby modulating TJ structural dynamics. As a principal structural and functional TJ protein, Occludin participates in barrier function regulation. Studies have demonstrated that the claudin protein family, another critical transmembrane protein in TJs, is implicated in tight junction complex formation in a DSS-induced inflammatory bowel disease mouse model. Claudin family members exhibit diverse distributions and functions across tissues, displaying tissue specificity and playing a pivotal role in barrier formation and selective permeability regulation.

Among them, the molecular weight of claudin-2 is about 24.5KD. During normal conditions, it is expressed in a minimal amount in the epithelial cell membrane of the crypt, creating a selective cation channel that is responsible for transporting cations through the cell bypass. The claudin-2 protein is also known as a pore-forming protein, and its increased expression can weaken the tight junction stability [11]. Most studies have shown that claudin-2 is highly expressed in crypt epithelial cells of mice with DSS-induced inflammatory bowel disease [12,13,14], causing an increasing of intestinal epithelial cell bypass permeability and damage to the mucosal barrier [15]. Similar to the above research results, the expression of claudin-2 in mouse colon tissue was significantly increased after DSS intervention. However, GB1 can regulate TJs by down-regulating claudin-2 expression and improve epithelial barrier function. In epithelial cells, one end of ZO-1 is connected with Occludin and claudin-2 in the cytoplasm, and the other end is combined with cytoskeletal components such as actin and stress fibers, and the cytoplasmic protein and the cytoskeletal system are stably connected together [16]. Our experimental results showed that the mRNA and protein expression of ZO-1 were significantly decreased in DSS-induced mouse UC model, however, after being prevented by GB1, the expression of ZO-1 was up-regulated, thereby improving the function of the epithelial barrier. This is consistent with the research results of Tong LC [17].

More and more evidences have proved that the pathogenesis of DSS-induced UC model is very similar to the humans’ [18]. There are too many inflammatory factors and the activation of immune cells such as neutrophils and macrophages during the onset. The pathogenesis of UC can be mediated by pro-inflammatory factors such as IL-1, IL-6, IL-8, and TNF-*α*. In inflammatory bowel disease, the expression levels of pro-inflammatory factors like IL-6 and TNF-α are increased and NF-κB are activated [19,20,21], revealing that the NF-κB signaling pathway and anti-inflammatory methods to protect the colon tissue from damage caused by excessive inflammatory response also play an important role in the body’s life activities. However, our study showed that DSS intervention caused the destruction of the colonic intestinal structure and produced excessive inflammation. DSS-induced colon inflammation can be drastically reduced by GB1 and the degree of colon damage can be significantly improved, on the other hand.

In addition, studies have reported that immune factors dominate in the pathogenesis of UC, the NLRP3 inflammatory body has become a vital target for the research of UC [22]. The mRNA-seq sequencing results of this study also showed that GB1 can regulate the NLRP3 inflammatory body signaling pathway.

In short, GB1 has a protective effect on DSS-induced colon injury. It reduces the production of IL-6, TNF-α and other inflammatory factors, and protects the intestinal mucosal barrier function through inhibiting the activation of NF-κB.

The receptor protein NOD-like receptor protein 3 of the NLRP3 inflammatory body belongs to the NLRs family. It is usually recruited and activated by the N-terminal domain, that is, the N-terminal Caspase, or the thermal protein domain (PYD). The repeated sequence of leucine is composed of the middle NACHT domain (NOD domain). In order to theoretically prove that GB1 has an activity regulating function, we first studied the binding site of GB1 and NLRP3-PYD through molecular docking. Molecular docking studies show that GB1 and protein residues ALA 65, ALA 69, LEU 12, and LYS 7 form hydrophobic interactions in different directions, and GB1 and NLRP3-PYD bind to each other.

Many studies have shown that the NLRP3 inflammatory body plays an important role in the pathogenesis of UC. Under normal circumstances, the expression of NLRP3 is too low. When inflammatory bowel disease occurs, the intestinal permeability increases and the mucosal barrier is destroyed, resulting in the production of microorganism and several pro-inflammatory factors. At the same time, the expression of NLRP3 and IL-1β precursors will increase, which leads to a series of subsequent activation reactions [23,24]. The activation of NLRP3 inflammatory cells can be inhibited to treat UC, as many related studies have found. In DSS-induced mouse colitis models, inhibition of mRNA expression of caspase-1 or IL-1β gene can alleviate colitis [25,26]. The study found that after DSS induction, macrophages lacking the NLRP3 encoding gene will affect the secretion of IL-1β, which indicates that the activation of caspase-1 is closely related to the NLRP3 inflammatory body pathway. In addition, Nlrp3^-/-^ mice induced by DSS showed lower colon inflammation and lower expression levels of inflammatory factors than wild-type mice [27]. Our research results also showed that the degree of weight loss, colon shortening and colon damage in Nlrp3^-/-^ mice are lower than those in WT mice after DSS induction. Many NLRP3 inflammatory body inhibitors have shown good therapeutic effects in animal models of NLRP3-related diseases, such as MCC950, CY-09, resveratrol, Rabdosia rubescens etc. Among them, MCC950 is the most widely used NLRP3 inflammatory body inhibitor. Subsequent studies have found that MCC950 has a good therapeutic effect on NLRP3 related diseases such as enteritis and atherosclerosis. The compound MCC950 can significantly inhibit the release of the pro-inflammatory cytokine IL-1β and involve in the inflammatory effects caused by NLRP3 inflammatory bodies [28]. Our results are consistent with the opinion of this article, the level of NLRP3 inflammatory bodies in the colon tissue of wild-type mice in the positive drug group was significantly reduced. DSS was employed in this study to cause intestinal inflammation in mice. Drinking DSS solution freely for 7 days to induce an experimental model similar to UC can cause diarrhea, rectal bleeding, shortening of the colon, ulceration, neutrophil infiltration, and a variety of the production of pro-inflammatory factors. The research results showed that the mRNA and protein expression levels of NLRP3, ASC, caspase-1, and IL-1β in the DSS model group were significantly increased. GB1 can reduce the mRNA and protein expression levels of NLRP3, ASC, caspase-1, and IL-1β in the tissues of colitis mice, but these effects of Nlrp3^-/-^ mice are abolished, indicating that GB1 has the ability to inhibit the activation of NLRP3 inflammatory bodies, reduces the production of pro-inflammatory factors and improves t-he inflammation of the colon of mice. At the same time, compared with wild-type mice, Nlrp3^-/-^ mice show a lower susceptibility to UC.

## 4. Materials and Methods

### 4.1. Drugs and Materials

Garcinia kola nuts were obtained from Nigeria (Africa) in 2017 and identified by Professor Gang Hao of South China Agricultural University (Guangzhou, China). The voucher specimens were deposited at the Artemisinin Research Center, Guangzhou University of Chinese Medicine. GB1 was isolated as previously described [10] and identified using HPLC, ^1^H-NMR, and ^13^C-NMR.The yield and purity were determined to be approximately 1.0 and 99.6%, respectively. Sulfasalazine pyridine (SASP) was purchased from Maclean (Bijing, China, batch number: S838221). Fluorescein isothiocyanate dextran (FITC-dextran 4000) was acquired from Xi’an Ruixi Biotechnology Co., Ltd., (Xi’an, China). Dextran Sulfate Sodium (DSS) was bought from MP Biomedicals, LLC. (Beijing, China, lot number: 160110). Myeloperoxidase kit (MPO) was purchased from Nanjing Jiancheng Bioengineering Institute (Nanjing, China). RNAiso Plus mRNA extraction kit was purchased from Beijing Solable Corporation (Beijing, China). The 5×All-in-One RT Master Mix reverse transcription kit was purchased from Abcam (Shanghai, China). SYBR Green Master Mix Amplification Kit was purchased from Thermo Fisher Scientific (Shanghai, China). RIPA cell lysate, color pre-stained protein marker, PierceTM Lane Marker R9educing Sample Buffer was purchased from Therm Fisher Scientific, (Shanghai, China). Protease inhibitor (PM-SF), protein quantitative (BCA) kit and SDS-PAGE gel rapid preparation kit were purchased from Beijing Dingguo Changsheng Biotechnology Co., Ltd., (Beijing, Chian). NF-κB p65, phospho-NF-κB p65, rabbit monoclonal antibody ZO-1, rabbit monoclonal antibody CD11b, rabbit monoclonal antibody Claudin2, rabbit monoclonal antibody Occludin, β-actin, goat anti-rabbit secondary antibody were purchased from abcam (Shanghai, China). ECL chemiluminescent color developing solution was purchased from BIO-RAD (Shanghai, China). 0.45 µm PVDF film was purchased from Merck Millipore (Shanghai, China). Bovine Serum Albumin (BSA) was purchased from Bovogen Biologicals Biological Products Co., Ltd. (Melbourne, Australia), IL-6 kit and TNF-α kit were purchased from Andy gene company (Beijing, China). 4% paraformaldehyde was purchased from Haoma Bio (Guangzhou, China). Isopropanol was purchased from Guangzhou Chemical Reagent Factory (Guangzhou, China). Glycine, SDS, Tris were purchased from Amresco (Shanghai, China). 10×TBST buffer was purchased from Beijing Solable Corporation (Beijing, China).

### 4.2. Animals and Treatment

All animal health and experimental studies was approved and conducted in accordance with its guidelines by the Animal Ethics Committee of Guangzhou University of Chinese Medicine. 8-week-old C57BL/6 male mice, weighing 18–22 g, were purchased from Beijing Viton Lihua Animal Experiment Center. (Certificate Number: SCXK (Guangdong): 2013-0002). Sixty SPF C57BL/6 male mice were fed adaptively for one week. Then the animals were randomly divided into 6 groups in ascending order of random numbers by using the Excel random method, including normal, DSS, SASP (100 mg·kg^−1^), DSS+GB1 (150 mg·kg^−1^), DSS+GB1 (75 mg·kg^−1^), DSS+GB1 (37.5 mg·kg^−1^). During the experiment period, mice in normal group were given distilled water and other groups were given 4% DSS after giving distilled water for 3 days. The drug groups were given SASP (100 mg·kg^−1^) or GB1 (150, 75, 37.5 mg·kg^−1^) by intragastric administrator once a day. All animals were given fluorescein isothiocyanate dextran (FITC-dextran 4000, 600 mg·kg^−1^) for 4 h on 10th day before sampling.

The NLRP3 knockout mice were given by professor Changhui Liu. 24 SPF C57BL/6 male mice (WT mice) were randomly divided into 4 groups by using the Excel random method, including normal, 4% DSS, MCC950 (10 mg·kg^−1^) and DSS+GB1 (150 mg·kg^−1^). Twenty-four NLRP3 knockout mice (Nlrp3^-/-^ mice) were randomly divided into 4 groups using the Excel random method, including normal, 4% DSS, MCC950 (10 mg·kg^−1^) and DSS+GB1 (150 mg·kg^−1^). During the experiment period, mice in normal group were given distilled water and other groups were given 4% DSS after giving distilled water for 3 days. The drug groups were given MCC950 (10 mg·kg^−1^) or GB1 (150 mg·kg^−1^) by intragastric administration once a day. Collect samples on 10th day.

### 4.3. Colitis Assessment

During the experiment, the scores of the disease active index (DAI) were conducted according to the weight, diarrhea and rectal bleeding of mice. Blood was collected from the mouse eyeballs and centrifuged at 12,000× *g* at 4 °C for 15 min after standing at room temperature for 0.5 h. Then collected the supernatant and stored at −20 °C. The mice were killed by cervical dislocation. Took out the entire colon immediately and measured its length. The colon was quickly cut into several parts and fixed in 4% paraformaldehyde solution, embedded in paraffin and subjected to conventional hematoxylin and eosin (H&E) staining sections. The sections were examined under an optical microscope (Leica) (Shanghai, China). H&E stained histological scores of colon specimens were evaluated blindly by two pathologists. Then put a small part of the colon into an EP tube containing 2.5% glutaraldehyde solution immediately. The colon was fixed with 1% osmium acid fixative solution for 3 h, embed with different proportions of pure acetone and embedding solution, stained with 3% lead citrate acetate and observed intestinal epithelial cell junction (TJ) by transmission electron microscopy. The remaining part of the colon was stored in the refrigerator at −80 °C for the next experiment.

### 4.4. Colonic Myeloperoxidase (MPO) Activity Detection

To observe the degree of colitis inflammation of mouse, this study evaluated tissue myeloperoxidase (MPO) activity linearly that related to neutrophil infiltration in inflammatory tissue. Detect the content in tissues according to the instructions of myeloperoxidase (MPO) kit. The MPO activity is expressed by U·g^−1^ and calculated according to the following formula.

MPO vitality (U·g^−1^) = (Detection OD value − Control OD value)/11.3 × sampling volume (g).

### 4.5. FITC-D Fluorescence Detection

The serum was diluted 10-fold with PBS. FITC-dextran 4000 was diluted 10-fold in the same ratio to make a standard. Took 200 μL samples in 96-well plate and measured the fluorescence expression.

### 4.6. Immunohistochemistry

Tissue sections were soaked in Poly-Lysine and dehydrated with xylene. Antigen was repaired by repairing kit containing 0.01M citrate buffer (pH = 6.0). Endogenous enzymes were inactivated by 3% hydrogen peroxide. 5% BSA blocking solution was used to block. Add appropriately diluted primary antibody and incubate at 4 °C overnight. Biotinylated goat anti-rabbit IgG was added dropwise and incubated at 37 °C for 30 min. The DAB kit was used to detect the signal. Observe and collect pictures with a microscope after dehydrating and fixing the coverslips.

### 4.7. Determination of Inflammatory Cytokines in Colitis

The mouse colon segments were homogenized in ice-cold PBS. After centrifugating at 3000× *g* at 4 °C for 10 min, the inflammatory cytokines (such as TNF-*α* and IL-1*β*) in the supernatant of homogenate were quantified using EL-ISA kit designated mouse inflammatory cytokines (Cusabio, Houston, TX, USA) according to the manufacturer’s instructions and guidelines. The results are expressed in pg·g-1 of the tissue in each sample. The experiment was conducted according to the kit instructions and the absorbance of each well was measured at a wavelength of 450 nm.

### 4.8. RNA Extraction and Quantitative RT-PCR Experiments

The total RNA in colon tissue was extracted using RNAiso Plus mRNA extraction kit. Nanodrop-2000c trace nucleic acid protein detector was used to detect the RNA concentration of the samples. The 5× All-in-One RT Master Mix reverse transcription kit was used for reverse transcription and the SYBR Green Master Mix amplification kit was used for target gene amplification. The following TaqMan probes were used: IL-1*β*, IL-6, TNF-*α*, ZO-1, Occludin, NLRP3, ASC, Caspase-1 and *β*-actin. Analyze the amplification curve to determine whether it is non-specific amplification after the amplification reaction is completed. Then we analyzed the dissolve curve and recorded the Ct value. Use *β*-actin as the internal reference gene, and calculate the difference of mRNA expression level of the target gene between each group using the 2^−△△^Ct method. Detailed primer sequences are listed in Table 1.

### 4.9. Western Blot Analysis

Samples from colon tissue of mouse (approximately 100 mg) were homogenized and incubated on ice for 30 min with RPIA lysate containing protease inhibitors and centrifuged at 12,000× *g* at 4 °C for 15 min. The supernatant was aspirated and tested the concentration of protein according to the instructions of the BCA kit. The proteins were separated by 6–12% separation gel according to the SDS-PAGE kit, and transferred to methanol-activated PVDF mem-brane. Then the membranes were incubated by 5% BSA blocking solution, and incubated overnight with primary antibodies target IL-1β (1:2000), IL-6 (1:2000), TNF-α (1:2000), ZO-1 (1:2000), Occludin (1:2000), NLRP3 (1:2000), ASC (1:2000) and Caspase-1 (1:2000). Then the blots were incubated with HRP-conjugated secondary antibody, and scanned after adding ECL exposure solution (A:B = 1:1). The gray value of the bands was analyzed with Image-Lab software (https://www.bio-rad.com/en-us/product/image-lab-software?ID=KRE6P5E8Z, accessed on 19 March 2025) and Image J software (https://imagej.net/ij/, accessed on 19 March 2025) after image acquisition.

### 4.10. mRNA-Seq Experiment

DNA contamination was detected by agarose gel electrophoresis. RNA purity and RNA integrity were accurately detected by Nanophotometer spectrophotometer and Agilent 2100 bioanalyzer. Use statistical methods to compare gene expression differences under normal, DSS, and GB1 group. Find specific genes related to conditions, and then further analyzed the biology significance of these specific genes.

### 4.11. Molecular Docking

The structure of the GB1 was downloaded from the Pubchem database and minimized through the molecular mechanics program to obtain the most stable structure. The three-dimensional crystal structure of NLRP3 protein was downloaded from the RCSB PDB database. The protein structure was modified by SYBYL software (https://sybyl.com/, accessed on 19 March 2025), such as hydrogenation, side chain repair, charge addition, extraction of embedded ligands, etc. And the protein’s docking active site was determined according to the location of its ligand. The total-Score and C-Score values are mutually referenced to evaluate the effect of molecular docking, and the optimal protein was selected and stored for preparation of the next molecular docking. Use the Surflex-Dock program of SYBYLX2.2.2 software to flexibly dock the optimal proteins in GB1 and NLRP3. The result and analysis of the connection are realized in Discovery Studio Visualizer.

### 4.12. Statistical Processing

The data was analyzed using SPSS20.0 statistical analysis software. The measurement data that conformed to the normal distribution were expressed as mean ± standard deviation ($\bar{x}\pm s$). One-way analysis of variance (One-way ANOVA) was used for comparison between groups. LSD method was used to compare the homogeneity of variance between two groups. Dunnett’s test was used between groups with uneven variance. *p* < 0.05 indicates that the difference is statistically significant. Graphpad Prism 9.0 software was used to deal with the output.

## 5. Conclusions

Overall, this is the first study to demonstrate the protective effect of GB1 on DSS against ulcerative colitis caused by DSS in murine models. The underlying molecular mechanisms of GB1 appear to involve the suppression of NLRP3 inflammasome activation, reduction in pro-inflammatory mediators, and the mitigation of DSS-induced ulcerative colitis. The study indicated that GB1 might serve as a natural NLRP3 inhibitor, thereby providing a new strategy for alternative colitis treatment.

## Figures and Tables

**Table 1:** Primer Sequence.

Primer	Sequences	Product Size
IL-1β	F1: 5′-GTTTCTGCTTTCACCACTCCA-3′ R1: 5′-GAGTCCAATTTACTCCAGGTCAG-3′	230 bp
IL-6	F1: 5′-CTGCAAGAGACTTCCATCCAG-3′ R1: 5′-AGTGGTATAGACAGGTCTGTTGG-3′	131 bp
TNF-α	F1: 5′-CAGGCGGTGCCTATGTCTC-3′ R1: 5′-CGATCACCCCGAAGTTCAGTAG-3′	89 bp
ZO-1	F1: 5′-GCCGCTAAGAGCACAGCAA-3′ R1: 5′-TCCCCACTCTGAAAATGAGGA-3′	134 bp
Occludin	F1: 5′-TTGAAAGTCCACCTCCTTACAGA-3′ R1: 5′-CCGGATAAAAAGAGTACGCTGG-3′	129 bp
NLRP3	F1: 5′-GCCTACAGTTGGGTGAAATGTAC-3′ R1:5′-ACAAGCCTTTGCTCCAGACC-3′	259 bp
ASC	F1:5′-GAAATACATCCCTACTTGGTG-3′ R1:5′-ATGTTTGGTATATGTTCTACCAC-3′	201bp
Caspase-1	F1:5′-GAGCTTCAATCAGCTCCATCAG-3′ R1:5′-CTTGAGGGTCCCAGTCAGTCC-3′	138 bp
β-actin	F1: 5′-GTGACGTTGACATCCGTAAAGA-3′ R1: 5′-GCCGGACTCATCGTACTCC-3′	245 bp

## Data Availability

All data generated or analyzed during this study are included in this article.
